# Research on the purchase intention of museum digital cultural and creative products based on value adoption model

**DOI:** 10.1038/s41598-025-02140-6

**Published:** 2025-05-25

**Authors:** Mengen Gu, Tongtong Zhao

**Affiliations:** 1 School of Art, Hunan University of Information Technology, Maotang Industrial Park, Xingsha Economic Development Zone, Changsha, 410151 Hunan Province China; 2 School of Fine Arts, Hunan Normal University, 638 Lushan South Road, Yuelu District, Changsha, 410012 Hunan Province China

**Keywords:** Purchase intention, Museum digital products, Cultural and creative industries, Value-based adoption model, Consumer behavior, Psychology, Human behaviour

## Abstract

Museum digital cultural and creative products rejuvenate ancient treasures and promote the development of cultural and creative industries. With the surge in purchases over the past few years, investigating the underlying factors has become a compelling question. Therefore, based on the Value-Based Adoption Model, this paper explores the factors and intrinsic mechanisms influencing consumers’ purchase decisions regarding these products. The collected data were analyzed using SPSS for preliminary analysis, and structural equation modeling was conducted using AMOS to test the research hypotheses. Results showed: (1) Perceived benefits positively influence purchase intention and value. (2) Perceived sacrifices negatively influence purchase intention and value. (3) Perceived value positively influences purchase intention and mediates the effects of perceived benefits and sacrifices. (4) Social influence positively moderates the relationship between perceived value and purchase intention. Specifically, entertainment experience, cultural experience, and perceived value positively influence purchase intention, while perceived cost and perceived risk negatively impact it. Based on these findings, developers should enhance product playability and cultural significance. Relevant departments should employ various strategies to reduce consumers’ cybersecurity risks and copyright issues during shopping while also minimizing selling costs to attract more consumer attention and purchases.

## Introduction

In recent years, the rapid advancement of digital technologies has ushered in a new era for cultural and creative industries, particularly in the museum sector^1^. Museum digital cultural and creative products (MDCPs) have emerged as a groundbreaking approach to revitalize cultural heritage and engage modern audiences^2^. MDCPs refer to digital assets or products developed by museums that integrate cultural heritage and creativity^3^. These products not only provide consumers with innovative ways to experience and appreciate cultural artifacts but also represent significant economic opportunities for museums and the broader cultural sector^4^. They serve as crucial means of national soft power competition, garnering attention from countries worldwide^5^. According to *Research and Markets* reports, the global non-fungible token (NFT) market, which includes MDCPs, is projected to reach $173.7 billion by 2028, with a compound annual growth rate (CAGR) of 41.6% from 2023 to 2028^6^. NFTs, as blockchain-based digital assets characterized by uniqueness and non-interchange ability, have become widely utilized in the cultural and creative sectors, providing new opportunities for the collection, trading, and investment of digital content. Since 2021, museums have employed blockchain technology to create digital collectibles for specific cultural relics, establishing a novel model for MDCPs. In 2021, the Dunhuang Academy collaborated with the Only Art Platform to launch the Dunhuang Series NFTs, revitalizing ancient culture through a combination of dynamic and static forms^7^. In 2022, the Shanghai Museum partnered with the Topnod Platform to issue digital cultural and creative collectibles from its collection^8^, which received widespread acclaim. These phenomena highlight the increasing consumer demand for digital cultural experiences and the potential for museums to diversify their revenue streams through innovative digital offerings. Given the significant role of MDCPs in cultural dissemination, economic growth, and the enhancement of national soft power, studying consumer purchase intentions for these products is a valuable and timely research endeavor.

Current research on MDCPs can be categorized into three types based on perspective and theme. Product development and promotion models^9^, design principles^10^, and risks and governance in the context of the metaverse^11^. While these studies offer theoretical support for the operation and design of MDCPs, they lack sufficient exploration from a consumer perspective, particularly regarding the relationship between perceived value and purchase intention for digital cultural products. In today’s highly competitive market environment, museums must enhance their products and services more appropriately and visibly to increase customer purchases. Since purchasing decisions are based on consumers’ overall value judgments of a product or service^12,13^, understanding this evaluative process is crucial. To better comprehend consumers’ purchasing motivations, it is vital to examine the factors influencing their purchase intentions from the perspective of perceived value.

The most well-known model for explaining individual purchase or adoption behavior is the Theory of Planned Behavior (TPB), proposed by Ajzen and Fishbein^14^. To explain and predict people’s acceptance of information technology, Davis^15^ built on TPB to develop the Technology Acceptance Model (TAM). However, Kim et al.^16^ argued that TAM is limited in explaining the adoption of new technology, such as electronic virtual products. Instead, they proposed the Value-based Adoption Model (VAM), which is more suitable for explaining the dual role of technology users and service consumers. Over the past decade, VAM has gained attention in the research field of cultural and creative industries. VAM, as a theoretical perspective and analytical tool, provides researchers with a comprehensive and systematic framework to understand and interpret the complex factors behind consumer purchasing behavior^12^.

In the cultural and creative industries, consumer purchasing behavior is often driven by various factors. First, digital cultural products offer rich entertainment enjoyment^17^ and unique cultural experiences^18^, which constitute significant perceived benefits for consumers. Second, since these products are typically purchased via online payment, consumers also consider potential perceived risks and costs when evaluating them, such as the uncertainty of information security risks and the reasonableness of pricing^19^. These perceived sacrifice factors collectively influence consumers’ overall perceived value of MDCPs, thereby further affecting their purchasing decisions. Additionally, social influence is a factor that cannot be overlooked. Existing studies have shown that in virtual product purchase decisions, external influences such as social circles and others’ evaluations play a crucial role in shaping consumer behavior and perceived value^20^. Therefore, in addition to perceived benefits and sacrifices, social influence, as an external factor, may also significantly impact consumers’ decision-making processes. When analyzing consumer purchase intentions, it is necessary to comprehensively examine the effects of these antecedent variables. Given this, VAM is particularly well-suited for understanding consumer behavior in this industry, as it not only clarifies the role of perceived value in influencing consumer purchase intentions but also fully accounts for the benefits and sacrifices that impact purchasing decisions^21^.

Based on the above context, this study adopts the VAM to focus on exploring the impact of entertainment enjoyment, cultural experience, perceived risk, perceived cost, and social influence on consumer purchase intentions. By investigating the mechanisms of these factors, this study aims to provide valuable practical insights for museums and related practitioners, helping them better optimize products and services to enhance consumer satisfaction and purchase intentions. Specifically, in this study, we attempt to answer the following research questions: (1) Do entertainment enjoyment, cultural experience, perceived risk, and perceived cost influence perceived value and consumer purchase intention? (2) Does perceived value influence purchase intention? (3) Does perceived value mediate the relationship between perceived benefits/sacrifices and purchase intention? (4) Does social influence moderate the relationship between consumer perceived value and purchase intention?

The following section is a general literature review covering the concept of MDCPs, the theory of perceived value, and the current state of research on consumer purchase intentions. In “Hypotheses development” section, we describe our research model and hypotheses. In “Research methodology” section, we present our research methods. In “Data analysis and results” section, we discuss our findings, and in “Discussion and implications” section, we summarize the implications of this study for practice and theory, as well as its limitations.

## Literature review

### Museum digital cultural creative products

Currently, there is no consensus on the terminology for museum digital cultural and creative products. The existing literature uses various terms such as “museum digital cultural heritage”^22^, “digital cultural and creative products”^23^, and “museum cultural creative merchandise”^24^. For the sake of clarity and consistency, this research refers to them collectively as “Museum Digital Cultural Creative Products.” These products are culturally creative items developed based on museum resources, combined with digital technology and creative thinking. They reflect the cultural connotations of museums, fulfill cultural dissemination and educational functions, and possess commodity attributes^25^. Specifically, MDCPs encompass artifacts, artworks, historical documents, and other materials collected by museums, which are digitized for storage, display, and sale through online platforms or digital technology. These digitized collections may include high-definition images of artifacts, three-dimensional models, virtual IP characters, and other forms, enabling product owners to browse and learn about museum collections remotely via the Internet or specific applications. Typically, these products are designed to attract audience purchases, promote cultural education, and expand the influence of the museum through digital platforms^26^.

Horbatch^27^ affirmed the critical role of digital technology in advancing the development of MDCPs, noting that digital technology has revitalized museums, making MDCPs more accessible and relatable to the general public. Current research on MDCPs predominantly focuses on content creativity and design, product marketing and promotion, financial risk control, and copyright protection, analyzing the underlying historical and cultural connotations, creative element conception, marketing strategies and dissemination trends, development strategies, and industry status. For instance, Hu^28^ believes that supported by government policies and various museum development platforms, this field is currently experiencing rapid growth. She further argues that it is the responsibility of MDCPs developers to innovate traditional cultural and creative products using digital technology, build cultural identity, achieve value transmission, and even connect the entire cultural industry. Regarding the formulation of MDCPs marketing strategies, Wang et al.^29^ suggest that the marketing tactics for MDCPs should include clear market positioning, enhancing user engagement, reasonable pricing, reliance on the internet, and increasing community interaction.

Currently, many renowned museums have adopted various marketing strategies to promote consumer purchasing behavior, such as hosting online virtual exhibitions, launching limited edition digital collectibles^30^, and developing mobile applications for cultural and creative products^31^. These strategies not only enhance the brand influence of museums but also increase consumer engagement and purchase desire. Nevertheless, it must be acknowledged that the domestic MDCPs market, which is still in its initial development phase, presents several issues on the supply side that warrant attention and reflection. For instance, the presentation forms are relatively homogeneous^10^, leading to insufficient aesthetic appeal and entertainment value. Additionally, the cultural content lacks depth, and platform regulatory mechanisms remain inadequate, among other prominent problems^32^. These issues could potentially limit consumer choice and dampen purchase intentions. Therefore, it is necessary to thoroughly analyze these factors to determine the extent to which they influence consumers’ purchase intentions and perceived value. Wang^33^ confirmed that to increase the sales of cultural and creative products and enhance the influence of museums, it is necessary to understand the relationship between consumers’ purchase intentions and perceived value. Sun et al.^34^ argued that more theory-driven empirical research is needed to deepen the understanding of consumer behavior in emerging digital industries, to improve the quality and relevance of research, and to promote the sustained adoption of these products or services by consumers.

### Value-based adoption model

**Fig. 1:**
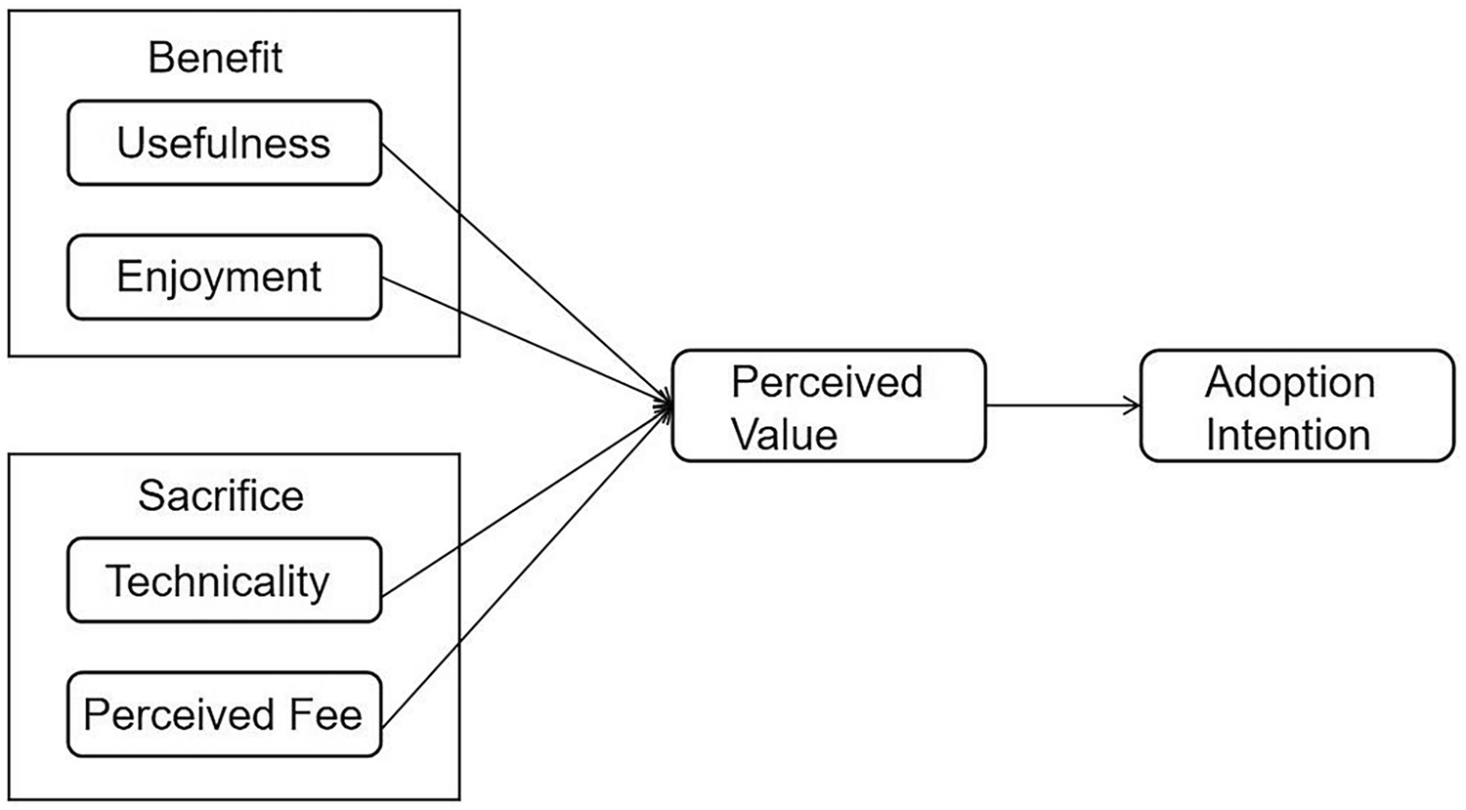
Value-based adoption model.

Research on perceived value began in the 1990s and has since become a focal point for scholars and entrepreneurs both domestically and internationally. Michael Porter^35^ pointed out that competitive advantage stems from the value a company creates for its customers. Woodruff^36^ defined customer value as the consumer’s perceived preference for and evaluation of product attributes, performance, and usage situations, which influence their intention to use the product. Based on previous studies, the VAM, developed by Kim et al.^16^, investigates consumer adoption of mobile commerce (see Fig. 1). This model emphasizes maximizing perceived value to explain the factors influencing individuals’ use of mobile internet. VAM posits a relationship between consumers’ intention to use and perceived value, which comprises perceived benefits and perceived sacrifices. Researchers widely use this model to assess consumer adoption of new products or services^37^, or integrate VAM with other models to study adoption intention across various products and services^21^. The theory of VAM not only focuses on consumers’ perceptions of a product’s functionality and usability but also emphasizes the entertainment and emotional experiences the product provides. This comprehensive approach explains consumers’ purchase intentions more holistically. Given that the VAM can fully capture the multidimensional value of museum digital cultural and creative products, it is well-suited to their complex consumption contexts. It also elucidates consumers’ subjective experiences and decision-making processes, making it particularly appropriate for studying consumer purchase intentions for MDCPs.

In recent years, an increasing number of scholars have utilized VAM to examine consumers’ adoption intentions regarding products or services in the cultural and creative industries. Table 1 summarizes previous research on perceived value in the context of digital virtual products or cultural and creative fields. Among these studies, Deng et al.^38^ combined VAM with existential theory and cognitive-affective behavior theory, introducing cultural identity as a mediating variable to explore the impact of digital museum experiences on visitors’ willingness to participate in on-site visits. Research results indicate that the more positive the cultural cognitive experience of visiting virtual museums, the stronger the visitors’ willingness to visit physical museums. However, this research did not adequately address the impact of perceived sacrifice. From the perspective of value perception, Fu^39^ designed a questionnaire and conducted data analysis on the cultural and creative products of the Guangxi Yao Ecological Museum, focusing on various value dimensions such as functional, social, cultural, and emotional value. The study proposed improvements for the development and design of the museum’s cultural and creative products. Although the research systematically explored various dimensions of perceived value, it fell short of clarifying the interrelationships among these dimensions and their comprehensive impact on purchase intentions. Based on field investigations and surveys of six major museums, Guo^40^ constructed a model of factors influencing the purchase intentions of museum cultural and creative products. The study concluded that consumer purchase intentions are mainly influenced by consumer satisfaction and perceived cost, with satisfaction primarily affected by perceived cultural value.

**Table 1:** Previous research on perceived value over virtual goods & cultural and creative contexts.

Research	Context	Antecedents of value	Consequences of value
Guo^40^	Consumption of cultural and creative products	Monetary price, perceived culture	Satisfaction, purchase intention
Shelvia and Prayitno^115^	Mobile payments	Convenience, social influence, benefit (usefulness, enjoyment), sacrifices(cost, risk)	Continuance intention
Fu^39^	Museum creative product design	Social value, emotional value, functional value	Purchase intention
Zhang^108^	Purchase of intangible cultural heritage handicraft products	Functional value, knowledge education value, social value, cultural identity	Purchase intention
Tseng and Wu^116^	Online learning platform	Instructional support, enthusiasm, preparedness	Learners’ positive reactions
Deng and Zhang^38^	Digital museuming experience	Perceived value, cultural identity, online experience	Willingness to visit on-site
Lai and Leong^117^	Adopt AR in online shopping	Ease of use, useful, personalized, innovative, highly engaging experience	Engagement and intention to use

In summary, existing research has primarily focused on physical museum cultural and creative products, with insufficient attention given to the product category of virtual digital creative products. Additionally, a significant body of research has explored the influence of perceived culture and perceived cost on consumers’ purchase intentions. However, the impact of entertainment experience, perceived risk, and social factors on perceived value and purchase intention has been largely overlooked.

According to the TPB, an individual’s behavioral attitudes influence their behavioral intentions^41^. In the context of cultural and creative product consumption, behavioral attitude can be viewed as the consumer’s attitude or awareness towards a particular cultural and creative product, which is directly influenced by perceived value. Individual behavioral intention can be interpreted as the consumer’s purchase intention. In the traditional realm of physical museum cultural and creative products, consumers mainly form purchase intentions by perceiving various values such as practical value, commemorative significance, material and craft^42^. However, in the case of MDCPs, digital cultural and creative products offer more personalized and immersive experiences, allowing consumers to experience the unique charm of cultural and entertainment in a virtual environment^28^. As a result, consumers form purchase intentions through values related to interactivity, cultural significance, and entertainment experience, thereby enhancing their behaviours to purchase. In light of this, examining the roles of entertainment experience and cultural experience is valuable for gaining deeper insights into the factors influencing purchase intentions.

With the widespread proliferation of social media, consumers engaging in online shopping are increasingly connected through their personal social networks. This connectivity enables them to discover, share, recommend, and review products, as well as exchange shopping information, knowledge, and opinions^43^. Consequently, they can obtain practical shopping advice and improve their shopping efficiency. According to source credibility theory, people are more easily persuaded by sources they deem credible^44^. Ma^45^ discovered that in the context of online shopping, information and recommendations from strong-tie contacts have a greater impact on purchase intentions than those from weak-tie contacts. Miao Ling^46^ pointed out that, compared to traditional cultural offerings in museums, digital collectibles—characterized by their digital nature, flexibility, and convenience—are more likely to appeal to the younger generation of consumers^47^, who are often more susceptible to peer influence^48^. Therefore, studying the role of social influence in the consumption of MDCPs is crucial. Additionally, information security risks also have a significant impact on purchase intentions, especially when it comes to digital products. It has been revealed that data privacy and consumer trust are “one of the most important issues in marketing right now^49^. There is an argument that privacy policies “serve as a basis for decision making for consumers^50^. Museum digital cultural and creative products typically rely on online platforms for transactions and delivery. Information security issues such as privacy breaches, payment security and data misuse, directly affect consumers’ trust in these platforms^51^. With the increasing frequency of cybersecurity incidents^52^, consumers are placing greater emphasis on the protection of personal information. A lack of trust can lead to a decline in consumer interest or even abandonment of the purchase. In summary, information security risks have become an indispensable factor in the purchase decisions of museum digital cultural and creative products.

This study specifically addresses MDCPs, a type of virtual cultural and creative product, while considering not only the commonly discussed factors like perceived culture and perceived cost related to museum cultural products but also incorporating entertainment experience due to the entertaining and highly interactive nature of digital virtual products^53^. Research has found that both external and internal factors can influence perceived value and behavioral intention^54^. While addressing the internal attributes of cultural and creative products, the paper also examines external factors affecting consumer purchase intention, such as perceived risk and social influence.

Based on this, our study combines the VAM theory with questionnaire surveys and structural equation modeling. This approach quantitatively explores how perceived value influences consumers’ purchase intentions and the underlying mechanisms during the consumption process of MDCPs. Some researchers believe that customer evaluations of products comprise both cognitive and emotional elements^55^, with purchases made to obtain utilitarian and hedonic benefits^56^. Incorporating Kim’s research on the VAM^16^, we suggest including entertainment experience and cultural experience as components of perceived benefits. Regarding perceived sacrifices, previous studies have categorized them into monetary and non-monetary costs^57,58^. Monetary costs refer to the price paid by the customer, while non-monetary costs include time, effort, and risks. Hence, we propose incorporating perceived fees and perceived information security risks as components of perceived sacrifices. Additionally, social influence is introduced as a moderating variable to investigate the impact of interpersonal communication behavior on the relationship between perceived value and purchase intention. The next section will elaborate on the related research hypotheses.

## Hypotheses development

### Perceived benefits

Perceived benefits can encompass tangible advantages, such as practical utility, as well as intangible benefits like perceived quality, enjoyment, and trust. Zeithaml et al.^59^ found that as users perceive higher benefits from a product or service, their perceived value also increases. From a social psychology perspective, the value of a product lies in its significance to the buyer’s community^60^. Specifically, products that carry particular meanings, such as entertainment and social culture, can enhance the social self-concept^61^. Given the characteristics of museum digital cultural creative products, we consider entertainment experience (EE) and cultural experience (CE) as components of perceived benefits.

**Fig. 2:**
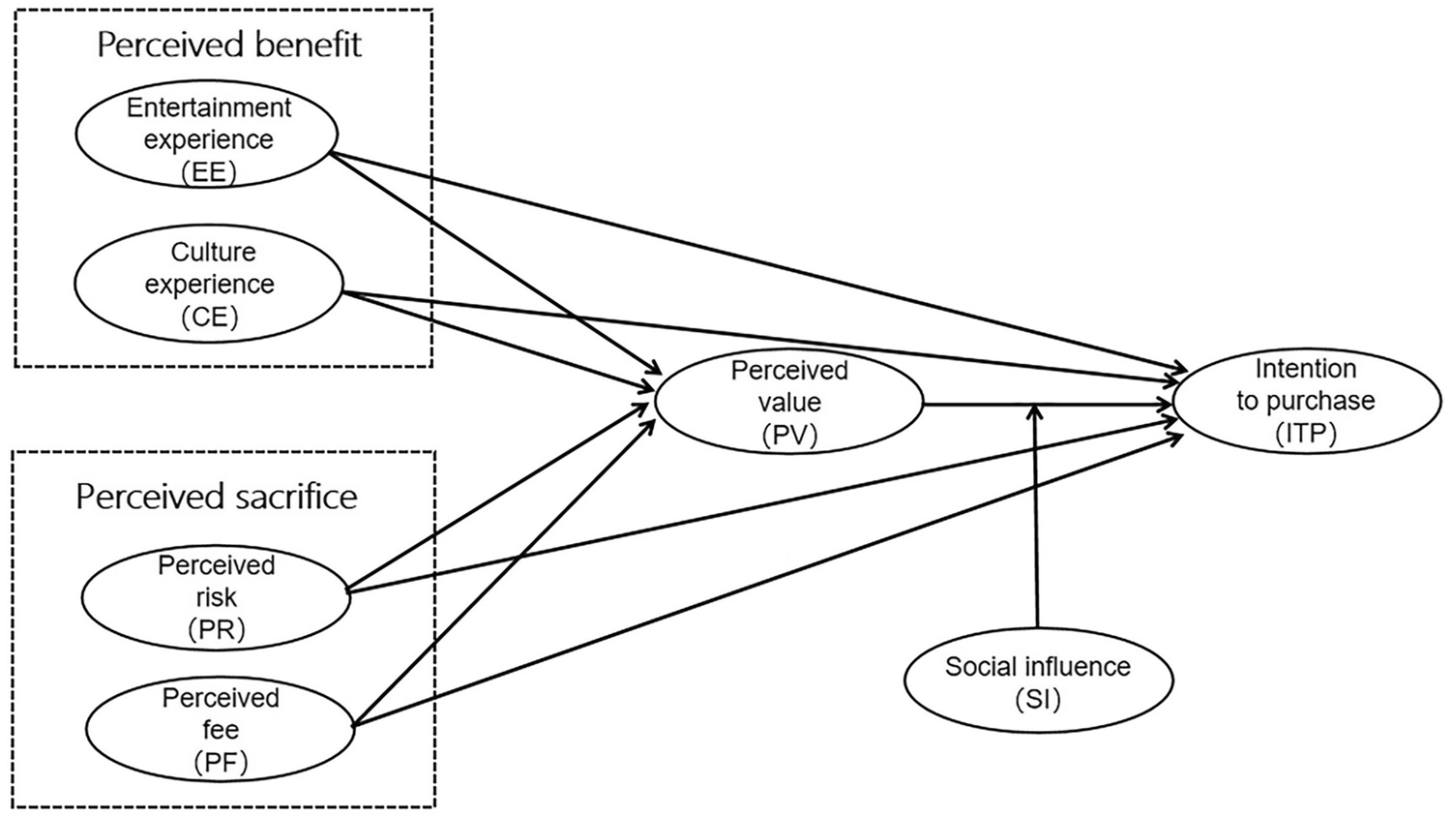
The hypothesized model.

In their study of website functionality and entertainment experience, Childers et al.^62^ found that emotional enjoyment has a greater impact on consumer perceived value and attitudes toward online shopping channels than information technology factors. Keeney contrasted consumers’ pre- and post-experiences of online shopping behavior and found that entertainment experience is a crucial dimension affecting attitudes toward online shopping. According to Yin^63^, consumers’ selection and purchase of museum cultural creative products often evoke certain emotional states or reactions. When consumers derive positive emotional satisfaction from museum cultural creative products, they are more likely to have higher purchase intentions. Gao et al.^64^ found that increasing the EE of cultural creative products positively impacts consumers’ perceived value, thereby affecting their purchasing attitudes. Based on this, the following hypotheses are proposed (see research model in Fig. 2):

#### H1a

*EE will positively affect consumers’ perceived value of MDCPs.*

#### H1b

*EE will positively affect consumers’ purchase intention for MDCPs.*

Cultural experience is a significant differentiator between museum cultural creative products and other products. The cultural heritage inherent in museum collections, combined with the creative design aspects of these products, provides unique market competitiveness^40^. Zhao et al.^65^ stated that consumers’ identification with national culture enhances their product evaluation, thereby increasing their purchase intention. Yang^66^ demonstrated that cultural values, such as pragmatic rationality, authoritative subordination, independence, and nostalgia, significantly positively influence the perceived value (PV) of cultural products from overseas museums. Functional, emotional, and social values mediate the relationship between cultural values and consumption intention of these cultural products. Su^67^ highlighted that consumers’ perceptions of the cultural significance, quality, and utility of Qiang embroidery clothing cultural and creative products, which bear intangible cultural heritage labels, have a significant positive impact. If consumers derive unique feelings from the cultural elements, symbols, or signs embedded in museum cultural creative products, their likelihood of purchase may increase. Based on this, the following hypotheses are proposed:

#### H2a

*CE value positively influences consumers’ perceived value of MDCPs.*

#### H2b

*CE value positively influences consumers’ purchase intention for MDCPs.*

### Perceived sacrifices

Perceived sacrifices encompass both monetary and non-monetary costs^68^. Monetary costs include the actual price of the product, typically assessed based on customers’ perceptions of the price paid. Non-monetary costs involve risks, time, effort, and other dissatisfactory experiences associated with the purchase and consumption of the product. Based on this rationale, we propose that perceived risk (PR) and perceived fee (PF) constitute the sacrifice components of perceived value. Perceived risk can be understood as the consumer’s subjective belief or perception of the potential for loss when pursuing a desired outcome^68^. Referring to the research by Kim et al.^69^, we define perceived risk in the context of MDCPs consumption as the potential information security risks perceived by customers, including risks of privacy leakage, online payment security risks, and other related aspects. Numerous exploratory studies have found that information security risks are a significant barrier to purchasing e-commerce products or services^70^. Jarvenpaa et al. believe that reducing the financial risk of shopping from online stores increases the likelihood of consumers making purchases from them^71^. Moreover, it has been proposed that perceived fee directly influences perceived value^58^. Marketing research indicates a negative relationship between perceived monetary price and perceived value^72^. Therefore, we hypothesize a negative relationship between perceived fees and overall perceived value: higher perceptions of fees are associated with lower perceptions of value. The following hypotheses are put forward:

#### H3a

*PR will negatively affect consumers’ perceived value of MDCPs.*

#### H3b

*PR will negatively affect consumers’ purchase intention for MDCPs.*

#### H4a

*PF will negatively affect consumers’ perceived value of MDCPs.*

#### H4b

*PF will negatively affect consumers’ purchase intention for MDCPs.*

### Perceived value and purchase intention

PV is viewed as a balance between the “give” and “get” aspects of a product^73^. From the perspective of consumer choice, consumers assess the value of a product by weighing all relevant benefits against the associated sacrifices^74^. As defined by Zeithaml^58^, perceived value arises from an individual’s comparison of the benefits gained and the sacrifices made following a purchase. This perception involves elements related to both consumer benefits and sacrifices. On one hand, the “benefit” component—what a consumer receives upon acquiring a product—includes perceived service quality and psychological advantages. On the other hand, sacrifices encompass both monetary and non-monetary costs, such as time, energy, effort, and inconvenience. To encourage consumers to purchase particular services or buy specific products again, these offerings must deliver value by either incorporating benefits or reducing sacrifices.

A customer’s intention to purchase (ITP) refers to their willingness to buy products or services from a specific shop or service provider. Purchase intention examines the reasons that cause customers to choose certain products or services^75^. The concept of purchase intention is also defined as a situation where consumers tend to buy a specific product under certain conditions^76^. Purchase intention is associated with customers’ value perceptions of MDCPs, their attitudes toward the product, and their shopping behaviors. Value perception reflects customers’ evaluation of the utility or benefits derived from the product. Attitude represents their positive or negative predisposition when making a purchase, influenced by factors such as prior experiences or peer recommendations. Behavior refers to observable actions, such as online browsing or shopping habits, which convey the likelihood of purchase. These interrelated factors make purchase intention an effective tool for predicting the purchasing process^77^. In this study, “purchase intention” primarily refers to consumers’ willingness to buy MDCPs or services, which is influenced by the comprehensive balance of perceived value and perceived cost. This balance affects consumers’ behavioral intentions and subsequently their actual purchasing behavior. Purchase intention includes activities such as paying attention to online flash sales, recommending products to others, and following new product releases. Purchase intention may change due to various factors, such as product price, perceived quality, and value. Regardless of the consumption situation, consumers aim to achieve maximum utility at the lowest cost. Thus, with limited resources, consumers will subconsciously judge the utility of goods to decide whether to make a purchase.

The higher the perceived value of a product, the more likely consumers are to pay for it. Eggert et al.^78^ discovered that consumers base their purchase decisions on maximizing perceived value, considering it the most crucial factor influencing purchase behavior. Similarly, Kleijnen et al.^79^ and Ko et al.^80^ found that perceived value significantly explains consumers’ purchase intentions. Therefore, perceived value influences purchase intention^81^. Su et al. through an empirical study, pointed out that perceived cultural experience not only directly influences consumers’ purchase intention but also indirectly affects it through perceived value^82^. Yang^66^ noted that when consumers engage with and select museum cultural and creative products, they form evaluations based on aspects such as appearance, functionality, cost-effectiveness, and cultural connotation. These evaluations directly influence their purchase intentions and may also indirectly affect their final purchase decisions through perceived value as a mediator. Based on this, we propose the following hypothesis:

#### H5

*PV positively influences the intention to purchase MDCPs.*

#### H5a

*PV mediates the relationship between EE and ITP.*

#### H5b

*PV mediates the relationship between CE and ITP.*

#### H5c

*PV mediates the relationship between PR and ITP.*

#### H5d

*PV mediates the relationship between PF and ITP.*

### Social influence

In addition to perceived value, social factors also play a critical role in shaping consumer behaviors, particularly in digital environments. Social influence refers to the extent to which consumers feel that important people, such as family and friends, believe they should use or purchase a certain type of product^83^. Wood and Hayes^84^ added that peer influence is more dominant in shaping behavior. The influence of reference groups can significantly determine an individual’s actions regarding certain behaviors. Indeed, much consumer behavior can be attributed to the influence of significant others. Considering that consumers purchasing museum cultural and creative products are predominantly young people^47^, who are often more susceptible to peer influence^48^, studying the role of social influence in the consumption of MDCPs is crucial. Li et al.^85^ believe that when consumers perceive that museum cultural creative products can bring additional social benefits, such as personal expression, identity affirmation, or gaining recognition or praise from others, they experience higher satisfaction and consequently develop stronger purchase intentions. Social influence has a significant moderating effect on the correlation between consumer attitudes and behavior^86^. Previous studies support this interaction effect^87^. Thus, we posit that the impact of consumers’ perceived value on ITP is greater when important others endorse or suggest that consumers should engage in the purchase. Based on this, the following hypothesis is proposed:

#### H6

*Social influence positively moderates the relationship between PV and ITP.*

## Research methodology

### Data collection and sample characteristics

This study targeted users who have either purchased museum cultural and creative products or have relevant experience with these products, such as browsing or interacting with MDCPs through online or offline platforms. The current museum cultural and creative products include both traditional physical items and digital products, with the latter primarily focusing on NFT replicas (3D models) of artifacts. In addition, many museums, in collaboration with other platforms or brands, have developed original digital IPs based on elements from their collections^77^, thereby enriching the forms and interactive experiences of these cultural products. To distinguish whether participants belong to the target group, the first section of the questionnaire included a question: “Have you purchased or browsed any museum digital cultural products, such as digital artworks of artifacts, NFT replicas, or virtual exhibitions?” If the answer was negative, the questionnaire was ended immediately. Additionally, the concept of museum digital collectibles was introduced in the first section of the questionnaire, along with some example images.to help participants correctly understand MDCPs. This study employed a convenience sampling method, which is a type of non-probability sampling. Data were collected through multiple strategies to ensure a diverse sample of respondents. The primary online survey platform used was Wenjuanxing, a popular and reliable online survey tool in China, widely employed by businesses, organizations, and researchers to gather data from internet users^88^. The survey questionnaire was distributed through group chats on three social media platforms: WeChat, QQ, and RED. Offline surveys were conducted at the Hunan Provincial Museum and the Changsha Museum, targeting visitors who browsed or purchased MDCPs. This combination of methods was employed to mitigate sampling bias and ensure a broad demographic representation.

The recruitment period for this study began on December 23, 2023, and ended on April 25, 2024. The study was conducted in strict accordance with ethical guidelines to ensure the protection of participants’ privacy and rights. Ethical approval was obtained from the Biomedical Research Ethics Committee of Hunan Normal University. Informed consent was obtained from all subjects and/or their legal guardian(s) prior to their voluntary participation in the study. Both online and offline surveys offered participants the chance to enter a prize-based lottery upon completion. Participants had the opportunity to receive a random cash reward ranging from 1 to 5 RMB. A total of 496 questionnaires were distributed, and 471 were returned. To enhance data quality, researchers screened the responses based on the content and duration of answers. After initial analysis, 46 invalid questionnaires—those with response times of less than 90 s or uniformly consistent answers—were excluded, resulting in 425 valid questionnaires and an effective rate of 90.23%.

The sample included participants of different ages, education levels, and incomes, supporting the generalizability of the conclusions. The surveyed participants were primarily young adults (aged 18–25), making up 79.3% of the sample, with a high level of education (bachelor’s degree and above), accounting for 80.3%. According to data released by iiMedia Research in its “2023 report on the consumer profile of China’s cultural and creative products industry”^89^, the majority of consumers in this sector are women, accounting for 63.2%, with nearly 90% of consumers aged between 19 and 40. Additionally, the “2024 Cultural and Creative Industry Report”^90^ published by Mob Research Institute (a globally leading data intelligence technology platform) highlights that younger demographics (including those born in the 1990s and 2000s) are the main consumer force, representing 79.2%, while highly educated consumers (with a bachelor’s degree or above) constitute 75.2%. Over 70% of respondents indicated that their spending typically falls within the range of 500 RMB or less. Based on this, overall, the research sample structure is reasonable, and the research data can be further analyzed using SPSS and Amos software. (Table 2).

**Table 2:** Demographic characteristics of the respondents.

Variable	Item	Frequency	Percentage (%)
Gender	Male	184	43.3
	Female	240	56.5
Age	18–25	337	79.3
	26–35	57	13.4
	36–45	16	3.8
	46–60	10	2.4
	Over 60	5	1.2
Education	High school or less	20	4.7
	Junior college	64	15.1
	Bachelor’s degree	291	68.5
	Master’s degree or above	50	11.8
Monthly income	Under ¥1000	43	10.1
	¥1000–5000	291	68.5
	¥5000–8000	44	10.4
	¥8000–10,000	26	6.1
	Over ¥10,000	21	4.9
Frequency of purchase	Less than 3 times	268	63.1
	4–6 times	131	30.8
	7–10 times	19	4.5
	10 times above	7	1.6
Product price	Under 50	286	67.3
	51–100	85	20
	101–500	39	9.2
	501–1000	13	3.1
	Over 1000	2	0.5

### Measurements and statistical analysis tools

All items in the questionnaire were validated and adapted from prior published studies (Table 3). The questionnaire was designed in six sections: The first section contains questions about demographic characteristics such as age, gender, education level, and income. The second section encompasses items for measuring perceived benefits, specifically entertainment experience (EE) and cultural experience (CE). Five items were used to measure EE, and four items evaluated CE, adapted from the Lee et al.^91^ and Liu et al.^92^ scale. Previous studies also confirmed the adequate psychometric properties of the scale^93^. The third section assesses perceived sacrifice, with four items dedicated to measuring perceived risk (PR) and another four items for measuring perceived fee (PF), adapted from Kim et al.^69^, McCloskey^94^, and Habibi et al.^72^. The fourth section evaluates social influence (SI) with five items based on Koenig-Lewis et al.^93^. The fifth section includes questions evaluating PV, with four items adapted from Gallarza et al.^95^ and Zeithaml et al.^58^ The final section includes questions to establish ITP, with four items based on Fernando et al.^96^ and Wang et al.^97^ Except for the items in the first section, all other items were measured on a 5-point Likert scale, where 1 indicates “strongly disagree” and 5 indicates “strongly agree.”

**Table 3:** Dimensions and question items of survey variables in the study.

Constructs	Measurement items	Source of the measurement
Entertainment experience (EE)	EE1. I feel very pleased when interacting with digital cultural and creative products from museums	Lee et al.^91^
	EE2. The design of digital cultural and creative products from museums is full of fun	
	EE3. Digital cultural and creative products from museums add enjoyment to my life	
	EE4. I find digital cultural and creative products from museums novel and interesting	
	EE5. Overall, experiencing digital cultural and creative products from museums is highly enjoyable	
Culture experiences (CE)	CE1. I believe digital cultural and creative products from museums reflect traditional cultural symbols	Liu et al.^92^
	CE2. I believe digital cultural and creative products from museums highlight regional cultural elements	
	CE3. I can gain historical and cultural knowledge from digital cultural and creative products from museums	
	CE4. I believe digital cultural and creative products from museums have unique cultural connotations	
Perceived risk (PR)	PR1. Compared to purchasing traditional physical cultural and creative products, buying digital cultural and creative products involves greater financial risk	Kim et al.^69^
	PR2. When purchasing digital cultural and creative products, I am concerned about the safety of personal data and financial privacy	
	PR3. I worry about providing financial information when purchasing digital cultural and creative products from museums	
	PR4. I worry about providing financial information when purchasing digital cultural and creative products from museums	
Perceived fee (PF)	PF1. The pricing of digital cultural and creative products from museums is relatively high	Habibi et al.^72^
	PF2. Excessive pricing is a barrier to my purchase of digital cultural and creative products from museums	
	PF3. Purchasing digital cultural and creative products from museums requires significant financial expenditure	
	PF4. Generally, my disposable income does not easily allow me to purchase the museum digital cultural and creative products I desire	
Social influence (SI)	SI1. People around me would recommend that I purchase digital cultural and creative products from museums	Koenig-Lewis et al.^93^
	SI2. People important to me would suggest that I buy digital cultural and creative products from museums	
	SI3. People important to me think I should purchase digital cultural and creative products from museums	
	SI4. Individuals who influence my behavior expect me to purchase digital cultural and creative products from museums	
Perceived value (PV)	PV1. Overall, purchasing digital cultural and creative products from museums is highly valuable	Gallarza et al.^95^; Zeithaml et al.^58^
	PV2. The value of purchasing digital cultural and creative products from museums exceeds the cost for me	
	PV3. Compared to the time I need to spend, purchasing digital cultural and creative products from museums is worthwhile	
	PV4. Compared to the effort I need to put in, purchasing digital cultural and creative products from museums is worthwhile	
	PV5.Compared to the fees I need to pay, purchasing digital cultural and creative products from museums offers good value for money	
Intention to purchase (ITP)	ITP1. In the future, I will consider purchasing digital cultural and creative products from museums	Fernando et al.^96^; Wang et al.^94^
	ITP2. I am willing to recommend high-quality digital cultural and creative products to my friends	
	ITP3. If given the opportunity, I am willing to purchase digital cultural and creative products from museums	
	ITP4. There is a high likelihood that I will purchase digital cultural and creative products from museums in the future	

To ensure the reliability and validity of the survey instrument, a pretest was conducted with a sample of 48 participants. Participants included individuals from different age groups and backgrounds to ensure the survey’s applicability and clarity. During the survey process, we paid special attention to participants’ feedback on the clarity of the questions and made minor adjustments to the wording based on their suggestions. Following data collection, we performed reliability and validity analyses. The results of the pretest showed that the survey had good internal consistency, with a Cronbach’s α coefficient greater than 0.75, indicating satisfactory reliability. Additionally, the KMO measure of sampling adequacy was greater than 0.7, and the Bartlett’s test of sphericity was significant (*p* < 0.01), confirming that the data were suitable for factor analysis. These results provided confidence in the validity and reliability of the survey items before the full-scale data collection. We followed a widely used two-step approach^98^ to analyze the data. This involved using SPSS 26.0 for descriptive statistical analysis and AMOS 24.0 for structural equation modeling (SEM) to conduct confirmatory factor analysis (CFA), model fitting, path analysis, and to examine relationships among variables. Additionally, SmartPLS 4.1 was employed for moderation effect analysis, as detailed in the next section.

## Data analysis and results

### Measurement model

The reflective measurement model should satisfy reliability and validity standards^99^. Cronbach’s alpha is commonly used to assess reliability, with higher values indicating greater consistency. A reasonable alpha coefficient of 0.7 is required for scale reliability, with values closer to 1 signifying higher reliability. Structural validity includes both convergent and discriminant validity. In this study, all variables had alpha coefficients above 0.8, indicating good reliability^100^. Additionally, all variables had standardized factor loadings greater than 0.6, AVE values over 0.5, and CR values above 0.7, demonstrating satisfactory convergent validity. Table 4 shows the reliability and validity analysis results for each study variable. Furthermore, the correlation coefficient of each variable with other variables did not exceed the square root of its AVE, indicating good discriminant validity^101^, as shown in Table 5.

**Table 4:** Reliability and convergent validity analysis.

Constructs	Items	Standardized factor loading	CR	AVE	Cronbach’s α
Entertainment experience (EE)	EE1	0.765	0.884	0.605	0.884
	EE2	0.761			
	EE3	0.763			
	EE4	0.788			
	EE5	0.812			
Culture experience (CE)	CE1	0.779	0.877	0.641	0.876
	CE2	0.781			
	CE3	0.779			
	CE4	0.862			
Perceived risk (PR)	PR1	0.822	0.881	0.649	0.881
	PR2	0.792			
	PR3	0.788			
	PR4	0.821			
Perceived fee (PF)	PF1	0.810	0.876	0.639	0.876
	PF2	0.771			
	PF3	0.822			
	PF4	0.795			
Perceived value (PV)	PV1	0.824	0.913	0.678	0.913
	PV2	0.836			
	PV3	0.831			
	PV4	0.792			
	PV5	0.835			
Intention to purchase (ITP)	ITP1	0.795	0.867	0.621	0.867
	ITP2	0.756			
	ITP3	0.784			
	ITP4	0.818

**Table 5:** Discriminant validity for the measurement model.

	EE	CE	PR	PF	PV	ITP
EE	**0.778**					
CE	0.275	**0.801**				
PR	− 0.284	− 0.272	**0.806**			
PF	0.293	0.284	− 0.318	**0.799**		
PV	0.214	0.126	− 0.223	− 0.347	**0.823**	
ITP	0.504	0.494	− 0.512	0.086	0.385	**0.788**

The square root of AVE is shown in bold on the diagonal.

### Structural model

#### Model fit analysis

**Fig. 3:**
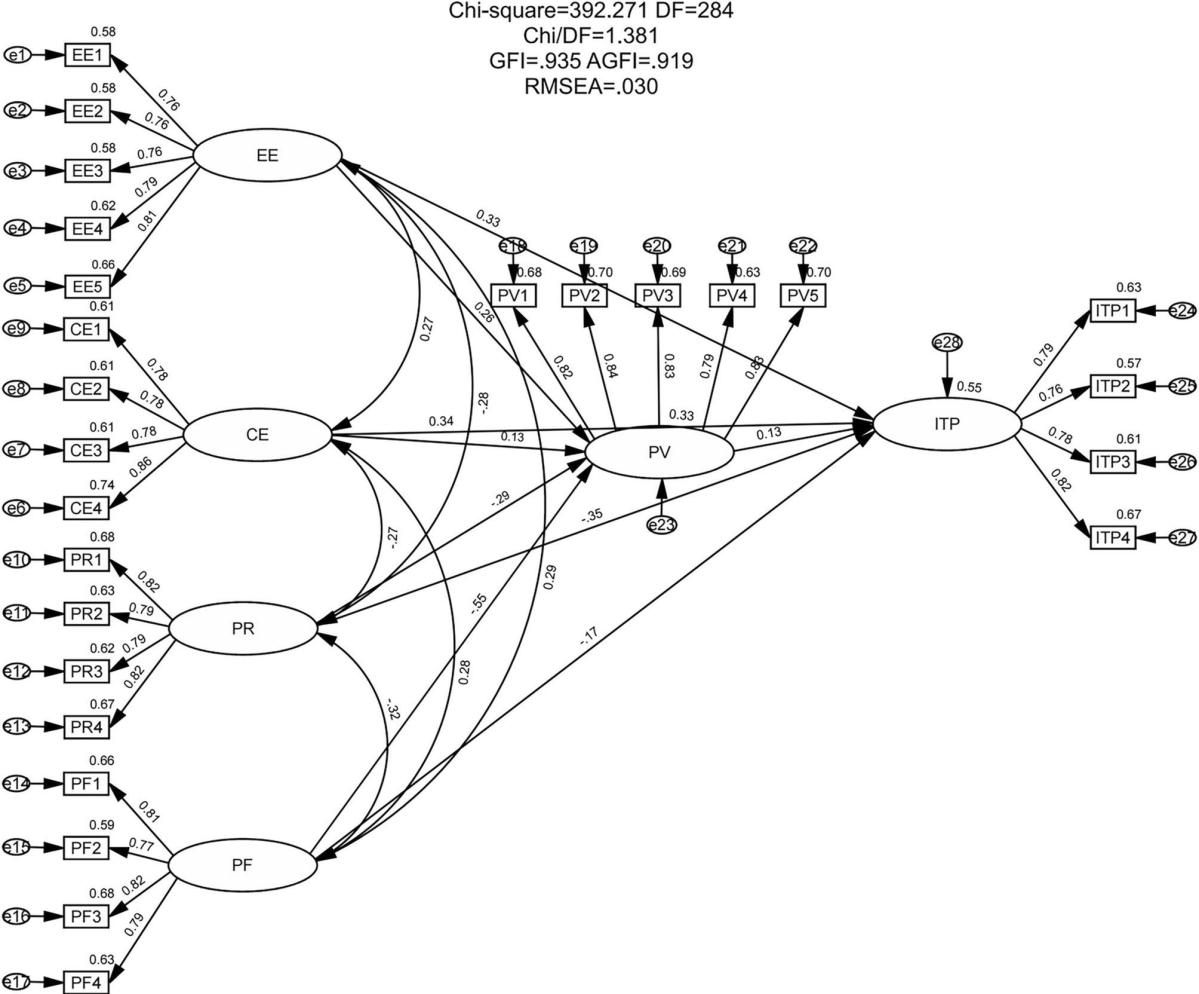
The structural model results.

Similar to the measurement model, the structural model was also found to possess satisfactory model fit indices, as shown in Fig. 3. The overall goodness-of-fit of the model was assessed using AMOS 24.0 prior to hypothesis testing. Model fit was analyzed using maximum likelihood estimation (MLE) and evaluated through several fit indicators: χ^2^/df value, root mean square error of approximation (RMSEA), goodness of fit index (GFI), adjusted goodness of fit index (AGFI), comparative fit index (CFI), incremental fit index(IFI), and Tucker-Lewis index (TLI). For large samples, the χ^2^/df criterion can be relaxed from 1–3 to 1–5; RMSEA below 0.05 indicates an excellent fit, while values between 0.05 and 0.08 indicate an acceptable fit. GFI, AGFI, CFI, IFI, and TLI values range from 0 to 1, with values above 0.9 representing an excellent fit. Parsimony normed fit index (PNFI) and parsimony comparative fit index (PCFI) values above 0.6 indicate an acceptable fit. Overall, the model fit is satisfactory, as presented in Table 6.

**Table 6:** Model fitness index values.

Index	Evaluation criteria	Research model	Pattern fitting
χ^2^	The smaller the better, *p* < 0.05	392.27 (*p* = 0.000)	Pass
χ^2^/df	1–5	1.381	Pass
RMSEA	< 0.08	0.030	Pass
GFI	> 0.9	0.935	Pass
AGFI	> 0.9	0.919	Pass
CFI	> 0.9	0.983	Pass
IFI	> 0.9	0.983	Pass
TLI	> 0.9	0.981	Pass
PNFI	> 0.6	0.823	Pass
PCFI	> 0.6	0.859	Pass

#### Hypothesis test

The structural equation model was constructed using AMOS 24.0 and tested to obtain standardized path coefficients (estimates), standard errors (SE), critical ratios (CR), and *p*-values. The standardized path coefficient reflects the change in Y when X changes by one standard deviation. The absolute value of the path coefficient indicates the correlation between variables. The critical ratio in the t-test is the t-value, and the *p*-value shows the significance of the path. A *p*-value below 0.05 generally indicates a valid hypothesis. The path test results confirm that all hypotheses are valid, as detailed in Table 7.

**Table 7:** Path test results.

Hypothesized paths	Estimate	S.E	C.R	*p*-value
H1:PV <---EE	0.257	0.061	4.765	***
H2:PV <---CE	0.133	0.057	2.543	*
H3:PV <---PR	− 0.289	0.052	− 5.268	***
H4:PV <---PF	− 0.552	0.061	− 9.158	***
H5:ITP <---EE	0.334	0.054	6.448	***
H6:ITP <---CE	0.339	0.05	6.811	***
H7:ITP <---PR	− 0.351	0.045	− 6.685	***
H8:ITP <---PF	− 0.174	0.055	− 2.968	*
H9:ITP < –-PV	0.133	0.049	2.471	*

*p < 0.05, ***p < 0.001.

#### Mediation models

To examine the indirect effects of the dependent variable via the mediators, we employed percentile bootstrapping and bias-corrected percentile bootstrapping with 5,000 samples at a 95% confidence interval^102^. Following the recommendations of Preacher et al.^103^, we calculated the confidence intervals for the standardized direct, indirect, and total effects of the hypothesized model, as shown in Table 8.

**Table 8:** Standardized direct, indirect, and total effects of the hypothesized model.

	Point estimate	Product of coefficients		Bootstrapping				
				Bias-corrected percentile 95% CI		Percentile 95% CI		Two-tailed significance
		SE	Z	Lower	Upper	Lower	Upper	
Total direct effects								
EE → ITP	0.383	0.05	7.660	0.291	0.483	0.289	0.483	**
CE → ITP	0.357	0.051	7.000	0.265	0.464	0.263	0.463	**
PR → ITP	− 0.337	0.043	− 7.837	− 0.431	− 0.259	− 0.422	− 0.250	**
PF → ITP	− 0.231	0.051	− 4.529	− 0.335	− 0.135	− 0.335	− 0.135	**
Indirect effects								
EE → ITP	0.036	0.015	2.400	0.01	0.072	0.007	0.068	*
CE → ITP	0.018	0.01	1.800	0.002	0.046	0.001	0.0410	*
PR → ITP	− 0.033	0.014	− 2.357	− 0.069	− 0.011	− 0.062	− 0.008	*
PF → ITP	− 0.068	0.028	− 2.429	− 0.127	− 0.02	− 0.124	− 0.017	*
Direct effects								
EE → ITP	0.347	0.051	6.804	0.246	0.443	0.249	0.447	**
CE → ITP	0.34	0.052	6.538	0.249	0.459	0.245	0.450	**
PR → ITP	− 0.304	0.045	− 6.756	− 0.404	− 0.222	− 0.398	− 0.215	**
PF → ITP	− 0.162	0.058	− 2.793	− 0.284	− 0.051	− 0.289	− 0.054	**

*p < 0.05, **p < 0.01.

The lower and upper bounds to test whether the indirect effects were significant, the bootstrap test results confirmed a positive and significant mediating effect of perceived value between enjoyment experience and purchase intention (standardized indirect effect 0.036, *p* < 0.05), and between culture experience and purchase intention (standardized indirect effect 0.018, *p* < 0.05). Additionally, the test confirmed a negative and significant mediating effect of perceived value between perceived risk and purchase intention (standardized indirect effect − 0.033, *p* < 0.05), and between perceived fee and purchase intention (standardized indirect effect − 0.068, *p* < 0.05).

#### Moderated models

**Fig. 4:**
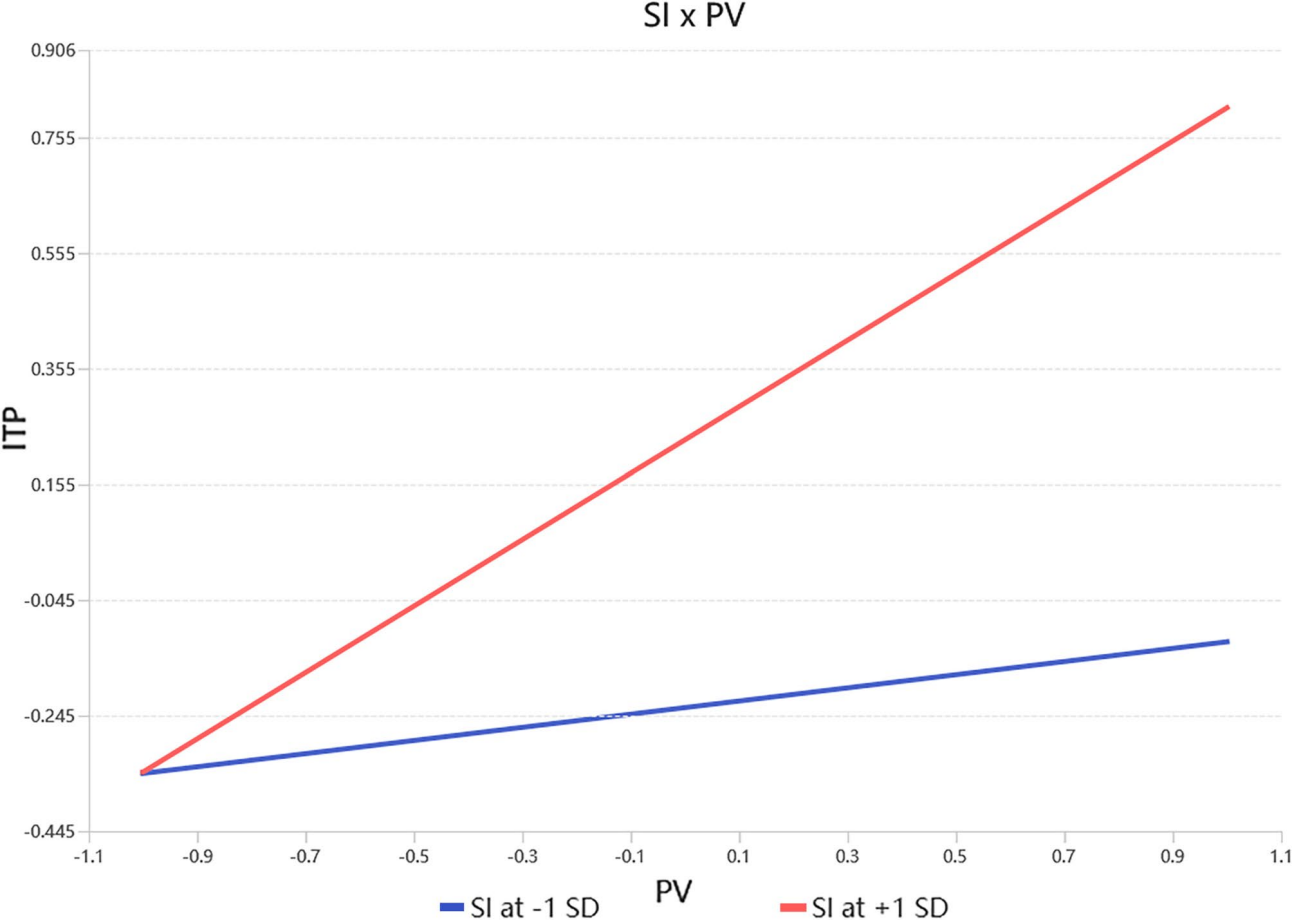
Simple slope analysis diagram.

The moderation analysis was conducted using SmartPLS, a widely used software for structural equation modeling^104^. This software was chosen due to its robustness in testing interaction effects and its ability to generate comprehensive outputs, including simple slope analysis and standardized path coefficients. The results, presented in Table 9, demonstrate that SI positively moderates the relationship between PV and ITP. Specifically, the interaction term (SI × PV) shows a significant positive effect on ITP (β = 0.230, T = 5.015, *p* < 0.001). This coefficient indicates that for every one-unit increase in SI, the positive effect of PV on ITP increases by 0.230 standardized units, suggesting that higher levels of SI amplify the impact of PV on ITP. Simple slope analysis further illustrates this moderating effect. As shown in Fig. 4, when SI is high (+ 1 SD), the relationship between PV and ITP is substantially stronger, as indicated by the steeper slope of the red line. Conversely, when SI is low (− 1 SD), the relationship remains positive but weaker, as reflected by the flatter slope of the blue line. Overall, these results emphasize the critical role of SI in enhancing the effect of PV on ITP.

**Table 9:** Moderation effect results analysis.

Path	Original sample	Mean	Std	T statistics	*p* values
PV – > ITP	0.344	0.348	0.041	8.316	***
SI – > ITP	0.232	0.236	0.045	5.187	***
SI × PV – > ITP	0.230	0.227	0.046	5.015	***

***p < 0.001.

## Discussion and implications

### Discussion

This study proposes a model of consumer purchase intention for MDCPs based on the VAM and develops a corresponding survey questionnaire. Structural equation modeling was used to analyze the primary factors influencing consumer perceived value for MDCPs and their impact levels. Understanding these influencing factors is of significant importance for advancing the development of MDCPs and enhancing their market competitiveness.

A key finding of this study is that perceived value has a positive impact on consumers’ purchase intention for MDCPs and plays a partial mediating role between perceived benefits/sacrifice and purchase intention. This finding is consistent with the findings of Gao et al.^64^. Although perceived value significantly affects purchase intention, the direct impact of perceived benefits and fee on purchase intention still exists. This may be because, when considering the purchase of MDCPs, consumers not only focus on the perceived value of the product but also take into account other factors such as brand influence, product uniqueness, and personal consumption habits. Therefore, perceived value acts as a partial mediator in this process, enhancing the influence of perceived benefits/fee on purchase intention.

Furthermore, this study demonstrates that customers’ perceptions of entertainment and cultural value in MDCPs significantly influence their perceived value, which subsequently impacts their purchase intention. Our research found that among the factors influencing consumers’ purchase intention of MDCPs, the impact of entertainment experience is more significant than that of cultural experience. In contrast to the research by Yang^66^ and Ning^105^ on museums’ traditional cultural and creative products. This discrepancy may be due to consumers focusing more on the entertainment or creative aspects of MDCPs compared to traditional physical cultural and creative products. Beyond statistical evidence, judgments based on Experience Economy Theory and Perceived Value Theory suggest that consumers consider not only functional value in their decision-making process but also place great importance on emotional experience and immersion^106^. Unlike static physical products, museum digital cultural and creative products offer greater interactivity, gamification mechanisms, and immersive experiences, which enhance consumers’ emotional engagement and enjoyment, thereby increasing brand loyalty and purchase intention. Additionally, the portability of these digital products (requiring no physical storage and allowing instant access) and their shareability cater to consumers’ desire for self-expression and identity construction through cultural consumption, fostering greater willingness for initial trial and repeat purchases.

Contrary to the outcomes generated by perceived benefits, perceived costs and perceived risks have a negative impact on perceived value, confirming Yilmaz and Zhang’s hypothesis^107,108^. Since the emergence of NFTs and MDCPs, both industry and academia have engaged in extensive discussions on associated risks. These include copyright issues related to digital collectibles^109^, the balance of interests among different stakeholders^110^, the protection of user rights, as well as technical, transactional, and financial risks^111^. Despite significant progress and advancements in the digital collectibles field over the past two years, and the gradual introduction of relevant risk management mechanisms^112^, challenges persist. This has led the public to remain cautious about the risks when purchasing virtual cultural and creative products. Consequently, risk assessment and prevention concerning MDCPs will remain important research directions for the foreseeable future. Moreover, the finding that the perception of cost negatively influences perceived value and purchase intention aligns with previous research on the cost perception of traditional physical museum cultural and creative products^32^. Both physical and digital forms of museum cultural and creative products are relatively expensive compared to similar ordinary goods, leading to a negative impact on consumer purchase intention. Therefore, development institutions need to consider the rational distribution of product pricing structures based on consumer consumption levels, striving to achieve a win–win goal of market acceptance, public satisfaction, and museum benefits.

Lastly, social influence moderates the relationship between perceived value and purchase intention positively. As social influence increases, the correlation between PV and consumers’ ITP strengthens. This result supports the viewpoint proposed by Yang et al.^113^ and Eagly and Chaiken^114^, who argued that a positive attitude may facilitate behavior when significant others approve. By purchasing MDCPs, consumers can join specific communities and gain group identity, providing unique social experiences. MDCPs not only establish a social connection between users and products but also facilitate interactions among users and interest groups.

Overall, the findings of this study support the core assumptions of the Value-based Adoption Model, particularly regarding the mediating role of perceived value and the impact pathways of perceived benefits and sacrifices. Additionally, this study highlights the unique characteristics of MDCPs, such as the significance of entertainment experience and the prominent role of social influence, thereby extending the applicability of VAM to the consumption of digital cultural and creative products.

### Implications

#### Theoretical implications

This study validates the perceived value theory by incorporating new variables into the widely accepted VAM and applying them in a novel context, which expands the applicability of existing theories. Moreover, the findings of this study offer valuable insights for research on MDCPs. It establishes a theoretical foundation for studying the impact of perceived value on consumer behavior, which is a core issue in the research on museum cultural and creative products. While the emergence of MDCPs brings more opportunities and challenges to the cultural and creative industries, it also adds complexity to the factors influencing consumer behavior. Therefore, the theoretical significance of this study is considerable, as it advances the understanding of the cultural and creative industries in the consumption domain. The methods and conclusions presented in this paper can serve as valuable references for researchers in related fields.

#### Practical implications

This study has significant practical implications for influencing consumers’ online purchasing behavior, particularly in the context of Chinese museums. Based on these research findings, the following recommendations can be made for museums and other relevant cultural e-commerce platforms, such as online marketplaces or digital distribution channels that offer MDCPs. These platforms play a critical role in enhancing accessibility, showcasing diverse offerings, and engaging broader audiences for MDCPs.

1. Enhancing the entertainment experience and cultural creativity of products is crucial. Research shows that stronger perceptions of entertainment and cultural experiences lead to higher perceived value. Therefore, the digital collection products of museums should focus on content and form innovation, which involves creating engaging narratives, incorporating diverse artistic styles, and utilizing advanced technologies like augmented reality (AR) or virtual reality (VR). These innovations enhance inclusivity and cater to the varying preferences of different demographic groups. MDCPs designers should explore contemporary entertainment and cultural trends, understand consumer preferences, and develop products that offer enhanced experiences. This increases consumer satisfaction, perceived value, and purchase intentions. Examples include launching cross-border collaboration digital collection blind boxes, co-branded digital collections, and interactive digital collections with social attributes. Linking digital collections with online and offline exhibitions, scene restorations, and collection protection can create immersive experiences and boost their attractiveness.
2. Enhancing social functions and expanding promotional channels is crucial for museums. This study found that social influence significantly moderates the relationship between PV and consumers’ ITP. Museums should leverage the multi-dimensional entertainment ecosystem and maintain high-quality content. Museums can establish exclusive platforms and engage with cultural communities through social interactions, gaming networks, and community-based activities. This approach bridges the gap between consumers and products, increasing attention and participation in digital collections. Expanding communication channels and extending the brand effect through new media forms like short videos and live streaming on platforms such as Sina, Weibo, RED, Douban, and Douyin (TikTok) is also essential. Marketing activities like themed holidays, discounts, lotteries, and referral bonuses can enhance interpersonal influence. Additional, continuously monitoring social media trends, rapidly adapting digital collection themes to align with contemporary cultural interests, regularly updating digital collectible designs to reflect current aesthetic and technological preferences, and engaging with emerging digital platforms. For example, promotional keywords such as “virtual culture guardian”, “cultural code breaker” and “digital heirloom hunter” can resonate with digital local audiences. Or during festivals like Spring Festival or Qingming Festival, museums could create digital collectibles incorporating zodiac themes, traditional symbols, or historical figures, similar to the Henan Museum’s successful “Collect Five Blessings” campaign with Alipay^114^.
3. Improve regulatory legal mechanisms to prevent infringement, fraud, and other security risks. Currently, gaps exist in the regulatory policies related to digital collections. As a result, consumers may have concerns such as cybersecurity risks when making purchases, which can negatively impact perceived value and purchase intention. To guide the healthy development of the digital cultural and creative industry, relevant government agencies should promptly establish a multi-level regulatory mechanism. This includes strengthening top-level design and timely promulgating or revising laws and regulations related to the development of digital collection applications. It is essential to clarify issues such as copyright, illegal appropriation, and fraud involved in the development and application of digital collections. Vigilance against speculative hype, ethical risks, and data security breaches is also necessary.
4. Enrich the price segments of products and reasonably delineate sales prices. Sales platforms for digital cultural and creative products should lower consumers’ perceived costs by diversifying price segments, thereby increasing their willingness to purchase. When setting product prices, it is important to follow general pricing principles while emphasizing the museum’s unique attributes and highlighting the additional value of creative products. Additionally, adherence to objective economic laws is essential to avoid arbitrary pricing. By designing product layouts around core price points, developing reasonable pricing strategies, and regulating the categories and quantities of products across various price segments, products can align with market acceptance and the expected psychological price points of specific consumer groups. This achieves a balance in supply and demand, yielding tangible economic benefits for museums.

### Limitations and future research

This study has several limitations. Firstly, the data are based on self-reported measures, which may be influenced by biases such as social desirability bias and inaccurate reporting of purchase behavior. Secondly, this study did not analyze responses according to specific survey modes, potentially leading to unexamined differences in the results. Future research on the impact of PV on consumers’ purchase intention for MDCPs can explore multiple directions. One potential avenue is the inclusion of additional variables, such as trust, user engagement, and technology acceptance, to expand the research model and provide a more comprehensive understanding of the mechanisms linking perceived value and purchase intention. Another direction involves considering diverse consumer characteristics, including age, educational level, and cultural background, to investigate their moderating effects on the relationship between perceived value and purchase intention. Moreover, researchers could conduct comparative analyses of different survey modes (e.g., online and offline questionnaires) to evaluate their potential influence on research outcomes.

## Data Availability

The datasets generated during the current study are available from the corresponding author on reasonable request.

## References

[1] Dalle Nogare, C. & Murzyn-Kupisz, M. Do museums foster innovation through engagement with the cultural and creative industries?. *J. Cult. Econ.* **45**, 671–704. 10.1007/s10824-021-09418-3 (2021).

[2] Massi, M., Marilena, V. & Yi, L. *Digital Transformation in the Cultural and Creative Industries. Production Consumption and Entrepreneurship in the Digital and Sharing Economy* 1–9 (Routledge, 2020).

[3] Guo, J. W., Tang, Y. L. & Meng, L. Research on the design and development of museum digital cultural and creative products in the context of art and technology. *Packag. Eng.* **45**, 107–112. 10.19554/j.cnki.1001-3563.2024.S1.013 (2024).

[4] Bai, B. On the present and future of digital collections in Chinese museums. *SHS Web Conf.* **157**, 04019. 10.1051/shsconf/202315704019 (2023).

[5] Peris-Ortiz, M., Gomez, J. A. & López-Sieben, M. *Cultural and Creative Industries: An Overview. Cultural and Creative Industries* 1–13 (Springer, 2019). 10.1007/978-3-319-99590-8_1.

[6] Research and Market. Non-fungible Token Global Market Report 2024—Research and Markets (n.d.) https://www.researchandmarkets.com/reports/5766985/non-fungible-token-global-market-report (2024).

[7] Dunhuang Art Institute Launches its First Dynamic Digital Art - Gansu Culture and Tourism - Silk Road (Dunhuang) International Cultural Expo, (n.d.). https://www.gswbj.gov.cn/a/2021/11/24/11734.html (2021).

[8] Shanghai Museum “Great Museum Plan” (n.d.). https://www.shanghaimuseum.net/mu/frontend/pg/article/id/I00004551 (2022).

[9] Aixin, B. & Zhou, Y. Exploring new innovative design approaches for cultural and creative development in local museums. *Packag. Eng.* **39**, 196–200. 10.19554/j.cnki.1001-3563.2018.20.032 (2018).

[10] Zhang, W. & Fan, K. Decoding “Homogenization” and exploring “New design”: Intelligent development and research of museum digital cultural and creative products in the AI era. *New Art.* **40**, 117–120 (2019).

[11] Wang, X. Risk deviations and governance regulations of museum digital collections in the metaverse perspective. *J. Southwest Univ. Natl. (Hum. Soc. Sci. Ed.)* **44**, 155–163 (2023).

[12] Shelvia, O., Prayitno, A., Rahim, R. & Sundjaja, A. Analysis of factors affecting consumer’s continuance intention to use mobile payments with a value-based adoption model (vam) approach. *Psychol. Educ.* **57**, 2883–2898 (2020).

[13] Regalado-Pezúa, O., Carvache-Franco, M., Carvache-Franco, O. & Carvache-Franco, W. Perceived value and its relationship to satisfaction and loyalty in cultural coastal destinations: A study in Huanchaco. *Peru. Plos One.* **18**, e0286923. 10.1371/journal.pone.0286923 (2023). 37527247 10.1371/journal.pone.0286923 PMC10393132

[14] Ajzen, I. The theory of planned behaviour: Reactions and reflections. *Psychol. Health.* **26**, 1113–1127. 10.1080/08870446.2011.613995 (2011). 21929476 10.1080/08870446.2011.613995

[15] Davis, F. D. Perceived usefulness, perceived ease of use, and user acceptance of information technology. *MIS Q.* **13**, 319–340. 10.2307/249008 (1989).

[16] Kim, H.-W., Chan, H. C. & Gupta, S. Value-based adoption of mobile internet: An empirical investigation. *Decis. Support Syst.* **43**, 111–126. 10.1016/j.dss.2005.05.009 (2007).

[17] Liu, Y. & Yin, J. Digital cultural and creative design of taohuawu woodblock new year paintings based on cultural translation. *Packag. Eng.* **43**, 326–334. 10.19554/j.cnki.1001-3563.2022.10.039 (2022).

[18] Kuang, J. & Zhan, S. The impact of cultural identity of digital collections on consumer purchase intentions: The mediating role of perceived value. *Manag. Sci. Res. Chin. Engl. Ed.* **11**, 70–74 (2022).

[19] Liu, Q., He, C., Chen, X. & Zhang, Z. NFT digital collections and digital cultural and creative development of glam institutions. *Libr. Constr.*10.19764/j.cnki.tsgjs.20230847 (2023).

[20] Alalwan, A. A. Investigating the impact of social media advertising features on customer purchase intention. *Int. J. Inf. Manag.* **42**, 65–77. 10.1016/j.ijinfomgt.2018.06.001 (2018).

[21] Lin, T.-C., Wu, S., Hsu, J.S.-C. & Chou, Y.-C. The integration of value-based adoption and expectation–confirmation models: An example of IPTV continuance intention. *Decis. Support Syst.* **54**, 63–75. 10.1016/j.dss.2012.04.004 (2012).

[22] Lynch, C. Digital collections, digital libraries & the digitization of cultural heritage information. *Microform Imaging Rev.* **31**, 131–145. 10.1515/MFIR.2002.131 (2002).

[23] Ai, Z., Chiu, D. K. W. & Ho, K. K. W. Social media analytics of user evaluation for innovative digital cultural and creative products: Experiences regarding dunhuang cultural heritage. *J. Comput. Cult. Herit.* **17**(42), 1–25. 10.1145/3653307 (2024).

[24] Wei, M., Chen, T. & Hsieh, Y. Study of cultural creative merchandises of museums and cultural heritage. *Adv. Graph. Commun. Print. Packag.* **543**, 447–451. 10.1007/978-981-13-3663-8_60 (2019).

[25] Tu, J.-C., Liu, L.-X. & Cui, Y. A study on consumers’ preferences for the palace museum’s cultural and creative products from the perspective of cultural sustainability. *Sustainability* **11**, 3502. 10.3390/su11133502 (2019).

[26] Chen, L. Technology, products, and value: Research on the application and development of digital cultural and creative products in china’s museum industry. *J. Educ. Media Res.* **02**, 57–59. 10.19400/j.cnki.cn10-1407/g2.2022.02.012 (2022).

[27] Horbatch, E. *Evaluating the Usage of Museum Digitized Collections*. https://jscholarship.library.jhu.edu/server/api/core/bitstreams/019c8b27-fff5-48c6-bfe8-c742aea987a4/content (2017).

[28] Hu, Y. Exploration of digital collection development in museums from the perspective of cultural value. *Southeast Culture.* 185–190. https://www.cnki.com.cn/Article/CJFDTotal-DNWH202303022.htm (2023).

[29] Wang, W. & Yao, W. Research on the development of digital cultural and creative products in museums. *Creat. Des. Source* **06**, 41–45+61 (2022).

[30] Cosovic, M. & Brkic, B. R. Game-based learning in museums-cultural heritage applications. *Information* **11**, 22. 10.3390/info11010022 (2020).

[31] Song, Y. & Li, M. Research on cultural and creative product development based on museum resources. *IOP Conf. Ser. Mater. Sci. Eng.* **452**, 022090. 10.1088/1757-899X/452/2/022090 (2018).

[32] Wang, J. Research on the impact of consumer perceived value on consumption behavior willingness for museum cultural and creative products in the new media era. In *Master’s thesis, Beijing Foreign Studies University.*10.26962/d.cnki.gbjwu.2023.000416 (2024).

[33] Wang, P. Study on the factors influencing consumption willingness of museum cultural and creative products. In *Master’s thesis, Minzu University of China.*10.27667/d.cnki.gzymu.2020.000528 (2021).

[34] Sun, B. & Du, H. Research on tourism cultural and creative product design based on consumption value theory. *Packag. Eng.* **43**, 333–340. 10.19554/j.cnki.1001-3563.2022.22.038 (2022).

[35] Porter, M. E. Technology and competitive advantage. *J. Bus. Strategy* **5**, 60–78. 10.1108/eb039075 (1985).

[36] Woodruff, R. B. Customer value: The next source for competitive advantage. *J. Acad. Mark. Sci.* **25**, 139–153. 10.1108/eb039075 (1997).

[37] Kim, D., Chun, H. & Lee, H. Determining the factors that influence college students’ adoption of smartphones. *J. Assoc. Inf. Sci. Technol.* **65**, 578–588. 10.1002/asi.22987 (2014).

[38] Deng, Y., Zhang, X., Zhang, B., Zhang, B. & Qin, J. From digital museuming to on-site visiting: The mediation of cultural identity and perceived value. *Front. Psychol.*10.3389/fpsyg.2023.1111917 (2023). 37034942 10.3389/fpsyg.2023.1111917 PMC10074853

[39] Fu, H. Optimization design of yao ethnic ecological museum cultural and creative products based on value perception. *Packag. Eng.* **43**, 386–393. 10.19554/j.cnki.1001-3563.2022.08.052 (2022).

[40] Guo, M. Research on perceived value and consumer intention of museum cultural and creative products. *Packag. Eng.* **39**, 223–227. 10.19554/j.cnki.1001-3563.2018.16.0375 (2018).

[41] Ajzen, I. The theory of planned behavior. *Organ. Behav. Hum. Decis. Process.* **50**, 179–211. 10.1016/0749-5978(91)90020-T (1991).

[42] Huang, H., Chen, H. & Zhan, Y. A study on consumers’ perceptions of museum cultural and creative products through online textual reviews: An example from palace museum’s cultural and creative flagship store. *Behav. Sci.* **13**, 318. 10.3390/bs13040318 (2023). 37102832 10.3390/bs13040318 PMC10135643

[43] Hu, X., Huang, Q., Zhong, X., Davison, R. M. & Zhao, D. The influence of peer characteristics and technical features of a social shopping website on a consumer’s purchase intention. *Int. J. Inf. Manag.* **36**, 1218–1230. 10.1016/j.ijinfomgt.2016.08.005 (2016).

[44] Appel, G., Grewal, L., Hadi, R. & Stephen, A. T. The future of social media in marketing. *J. Acad. Mark. Sci.* **48**, 79–95. 10.1007/s11747-019-00695-1 (2020). 32431463 10.1007/s11747-019-00695-1 PMC7222052

[45] Ma, L., Zhang, X., Ding, X. & Wang, G. How social ties influence customers’ involvement and online purchase intentions. *J. Theor. Appl. Electron. Commer. Res.* **16**, 395–408. 10.3390/jtaer16030025 (2021).

[46] Ling, M. Research on the development and design of digital collections in ethnic museums in China. *Guizhou Ethn. Stud.* **44**, 130–136. 10.13965/j.cnki.gzmzyj10026959.2023.01.022 (2023).

[47] Guo, L., Hang, Y. & Wu, J. A study on the differences of consumers’ emotional experience of cultural creative products in the palace museum of Beijing. *Cross-Cultural Des.* **14024**, 141–151. 10.1007/978-3-031-35946-0_12 (2023).

[48] Roberts, J. A., Manolis, C. & Tanner, J. F. Jr. Interpersonal influence and adolescent materialism and compulsive buying. *Soc. Influ.* **3**, 114–131. 10.1080/15534510802185687 (2008).

[49] Zhang, N., Wang, C., Karahanna, E. & Xu, Y. Peer privacy concern: Conceptualization and measurement. *MIS Q.* **46**, 491–530. 10.25300/MISQ/2022/14861 (2022).

[50] Zeng, F., Ye, Q., Yang, Z., Li, J. & Song, Y. A. Which privacy policy works, privacy assurance or personalization declaration? An investigation of privacy policies and privacy concerns. *J. Bus. Ethics* **176**, 781–798. 10.1007/s10551-020-04626-x (2022).

[51] Hassan, M. A., Shukur, Z., Hasan, M. K. & Al-Khaleefa, A. S. A review on electronic payments security. *Symmetry-Basel* **12**, 1344. 10.3390/sym12081344 (2020).

[52] Ho, F. N., Ho-Dac, N. & Huang, J. S. The effects of privacy and data breaches on consumers’ online self-disclosure, protection behavior, and message valence. *SAGE Open* **13**, 21582440231181396. 10.1177/21582440231181395 (2023).

[53] Papagiannidis, S., Pantano, E., See-To, E. W. K., Dennis, C. & Bourlakis, M. To immerse or not? Experimenting with two virtual retail environments. *Inf. Technol. People* **30**, 163–188. 10.1108/ITP-03-2015-0069 (2017).

[54] Rogers, E. M., Singal, A. & Quinlan, M. Diffusion of innovations. In *An Integrated Approach to Communication Theory and Research* 432–438 (Routledge, 2014).

[55] Dube, L. The power of affective reports in predicting satisfaction judgments. *Adv. Consum. Res.* **17**, 571–576 (1990).

[56] Babin, B. J., Darden, W. R. & Griffin, M. Work and/or Fun: Measuring hedonic and utilitarian shopping value. *J. Consum. Res.* **20**, 644–656. 10.1086/209376 (1994).

[57] Thaler, R. Mental accounting and consumer choice. *Mark. Sci.* **4**, 199–214. 10.1287/mksc.4.3.199 (1985).

[58] Zeithaml, V. Consumer perceptions of price, quality, and value - a means-end model and synthesis of evidence. *J. Mark.* **52**, 2–22. 10.1177/002224298805200302 (1988).

[59] Zeithaml, V. A., Verleye, K., Hatak, I., Koller, M. & Zauner, A. Three decades of customer value research: Paradigmatic roots and future research avenues. *J. Serv. Res.* **23**, 409–432. 10.1177/1094670520948134 (2020).

[60] Sheth, J., Newman, B. & Gross, B. Why we buy what we buy—A theory of consumption values. *J. Bus. Res.* **22**, 159–170. 10.1016/0148-2963(91)90050-8 (1991).

[61] Sweeney, J. C. & Soutar, G. N. Consumer perceived value: The development of a multiple item scale. *J. Retail.* **77**, 203–220. 10.1016/S0022-4359(01)00041-0 (2001).

[62] Childers, T. L., Carr, C. L., Peck, J. & Carson, S. Hedonic and utilitarian motivations for online retail shopping behavior. *J. Retail.* **77**, 511–535. 10.1016/S0022-4359(01)00056-2 (2001).

[63] Yin, D. Analysis of paths to enhance the fun and interactivity of museum cultural and creative products. *Cult. Relics Apprais. Apprec.* **7**, 81–84. 10.20005/j.cnki.issn.1674-8697.2023.07.021 (2023).

[64] Gao, L. & Zhang, M. Factors influencing consumers’ purchase intentions for intangible cultural heritage products and their mechanisms. *Econ. Manag. Res.* **39**, 126–135. 10.13502/j.cnki.issn1000-7636.2018.01.012 (2018).

[65] Zhao, S., Tu, Y. & Jiang, Q. The influence of cultural identity on consumer purchasing behavior. *J. Humanit. Arts Soc. Sci.* **7**, 788–809. 10.26855/jhass.2023.04.025 (2023).

[66] Yang, C. Research on the factors influencing overseas museum cultural and creative products consumers’ purchase intentions based on cultural values. In *Master’s thesis, Donghua University*. 10.27012/d.cnki.gdhuu.2019.000338 (2020).

[67] Su, J. The influence of perceived cultural identity on consumer purchase intention of ethnic cultural products: The mediating role of perceived value: A case study of Liangshan Yi embroidery cultural products. *Highlights Bus. Econ. Manag.* **9**, 222–232. 10.54097/hbem.v9i.9066 (2023).

[68] Pavlou, P. A. Consumer acceptance of electronic commerce: Integrating trust and risk with the technology acceptance model. *Int. J. Electron. Commer.* **7**, 101–134. 10.1080/10864415.2003.11044275 (2003).

[69] Kim, D. J., Ferrin, D. L. & Rao, H. R. A trust-based consumer decision-making model in electronic commerce: The role of trust, perceived risk, and their antecedents. *Decis. Support Syst.* **44**, 544–564. 10.1016/j.dss.2007.07.001 (2008).

[70] Rouibah, K., Al-Qirim, N., Hwang, Y. & Pouri, S. G. The determinants of ewom in social commerce: The role of perceived value, perceived enjoyment, trust, risks, and satisfaction. *J. Glob. Inf. Manag. JGIM.* **29**, 75–102. 10.4018/JGIM.2021050104 (2021).

[71] Jarvenpaa, S. L., Tractinsky, N. & Vitale, M. Consumer trust in an Internet store. *Inf. Technol. Manag.* **1**, 45–71. 10.1023/A:1019104520776 (2000).

[72] Habibi, A. & Rasoolimanesh, S. M. Experience and service quality on perceived value and behavioral intention: Moderating effect of perceived risk and fee. *J. Qual. Assur. Hosp. Tour.* **22**, 711 (2021).

[73] Dodds, W. B. & Monroe, K. B. The effect of brand and price information on subjective product evaluations. *Adv. Consum. Res.* **12**, 85 (1985).

[74] Kahneman, D. & Tversky, A. Prospect theory: An analysis of decision under risk. In *Handbook of the Fundamentals of Financial Decision Making* Vol. 4 99–127 (World Scientific, 2012). 10.1142/9789814417358_0006.

[75] Chang, T.-Z. & Wildt, A. R. Price, product information, and purchase intention: An empirical study. *J. Acad. Mark. Sci.* **22**, 16–27. 10.1177/0092070394221002 (1994).

[76] Morrison, D. G. Purchase intentions and purchase behavior. *J. Mark.* **43**, 65–74. 10.1177/002224297904300207 (1979).

[77] Talih Akkaya, D., Akyol, A. & Gölba Şışimşek, G. The effect of consumer perceptions on their attitude, behavior and purchase intention in social media advertising. *M U Iktis. Ve Idari Bilim. Derg.*10.14780/muiibd.384073 (2018).

[78] Eggert, A. & Ulaga, W. Customer perceived value: A substitute for satisfaction in business markets?. *J. Bus. Ind. Mark.* **17**, 107–118. 10.1108/08858620210419754 (2002).

[79] Kleijnen, M., de Ruyter, K. & Wetzels, M. An assessment of value creation in mobile service delivery and the moderating role of time consciousness. *J. Retail.* **83**, 33–46. 10.1016/j.jretai.2006.10.004 (2007).

[80] Ko, E., Kim, E. Y. & Lee, E. K. Modeling consumer adoption of mobile shopping for fashion products in Korea. *Psychol. Mark.* **26**, 669–687. 10.1002/mar.20294 (2009).

[81] Teck Weng, J. & de Cyril Run, E. Consumers’ personal values and sales promotion preferences effect on behavioural intention and purchase satisfaction for consumer product. *Asia Pac. J. Mark. Logist.* **25**, 70–101. 10.1108/13555851311290948 (2013).

[82] Su, S., Sun, C. & Chen, R. Cultural values, consumer perceived value, and purchase decision styles: A comparative study based on urbanization differences in china. *Nankai Bus. Rev.* **16**, 102–109 (2013).

[83] Dahl, D. et al. Social influence and consumer behavior. *J. Consum. Res.* **40**, iii–v. 10.1086/670170 (2013).

[84] Wood, W. & Hayes, T. Social influence on consumer decisions: Motives, modes, and consequences. *J. Consum. Psychol.* **22**, 324–328. 10.1016/j.jcps.2012.05.003 (2012).

[85] Li, Z., Shu, S., Shao, J., Booth, E. & Morrison, A. M. Innovative or not? The effects of consumer perceived value on purchase intentions for the palace museum’s cultural and creative products. *Sustainability* **13**, 2412. 10.3390/su13042412 (2021).

[86] Axsen, J. & Kurani, K. S. Social influence, consumer behavior, and low-carbon energy transitions. *Annu. Rev. Environ. Resour.* **37**, 311–340. 10.1146/annurev-environ-062111-145049 (2012).

[87] Conner, M. & McMillan, B. Interaction effects in the theory of planned behaviour: Studying cannabis use. *Br. J. Soc. Psychol.* **38**, 195–222. 10.1348/014466699164121 (1999). 10392450 10.1348/014466699164121

[88] Wang, L., Wong, P. P. W. & Zhang, Q. Travellers’ destination choice among university students in China amid COVID-19: Extending the theory of planned behaviour. *Tour. Rev.* **76**, 749–763. 10.1108/TR-06-2020-0269 (2021).

[89] iMedia Data Center. “Survey data on the development status and consumer behavior of china’s cultural and creative product industry.” (n.d.). https://data.iimedia.cn/data-classification/theme/49275257 (2024).

[90] MobTech. “2024 Cultural and creative industry report.” (n.d.). https://www.mob.com/mobdata/report/185 (2024).

[91] Lee, M.-C. & Tsai, T.-R. What drives people to continue to play online games? An extension of technology model and theory of planned behavior. *Int. J. Hum.-Comput. Interact.* **26**, 601–620. 10.1080/10447311003781318 (2010).

[92] Liu, L. & Zhao, H. Research on consumers’ purchase intention of cultural and creative products—Metaphor design based on traditional cultural symbols. *PLoS ONE* **19**, e0301678. 10.1371/journal.pone.0301678 (2024). 38739577 10.1371/journal.pone.0301678 PMC11090307

[93] Koenig-Lewis, N., Marquet, M., Palmer, A. & Zhao, A. L. Enjoyment and social influence: predicting mobile payment adoption. *Serv. Ind. J.* **35**, 537–554 (2015).

[94] McCloskey, D. Evaluating electronic commerce acceptance with the technology acceptance model. *J. Comput. Inf. Syst.* **44**, 49–57. 10.1080/02642069.2015.1043278 (2003).

[95] Gallarza, M. G. & Saura, I. G. Value dimensions, perceived value, satisfaction and loyalty: An investigation of university students’ travel behaviour. *Tour. Manag.* **27**, 437–452. 10.1016/j.tourman.2004.12.002 (2006).

[96] Fernando, G.-P., David, P.-C. & Sergio, A.-B. Effect of service experience, engagement and satisfaction on the future intentions of customers of a sports services. *Helion* **9**, e17850. 10.1016/j.heliyon.2023.e17850 (2023). 10.1016/j.heliyon.2023.e17850 PMC10345362 37455980

[97] Wang, Y., Lo, H.-P. & Yang, Y. An integrated framework for service quality, customer value, satisfaction: Evidence from china’s telecommunication industry. *Inf. Syst. Front.* **6**, 325–340. 10.1023/B:ISFI.0000046375.72726.67 (2004).

[98] Talwar, S., Dhir, A., Kaur, P. & Mantymaki, M. Barriers toward purchasing from online travel agencies. *Int. J. Hosp. Manag.* **89**, 102593. 10.1016/j.ijhm.2020.102593 (2020).

[99] Gironda, J. T. Review of advanced issues in partial least squares structural equation modeling. *J. Mark. Anal.* **12**, 108. 10.1057/s41270-023-00275-x (2024).

[100] Eysenck, S. Citation-Classic—The Measurement of psychoticism—A study of factor stability and reliability. *Curr. Contents Social Behav. Sci.*10.1111/j.2044-8260.1968.tb00571.x (1986). 10.1111/j.2044-8260.1968.tb00571.x 5706464

[101] Mason, C. & Perreault, W. Collinearity, power, and interpretation of multiple-regression analysis. *J. Mark. Res.* **28**, 268–280. 10.2307/3172863 (1991).

[102] Taylor, A. B., Mackinnon, D. P. & Tein, J. Y. Tests of the three-path mediated effect. *Organ. Res. Methods.* **11**, 241–269. 10.1177/1094428107300344 (2008).

[103] Preacher, K. J. & Hayes, A. F. Asymptotic and resampling strategies for assessing and comparing indirect effects in multiple mediator models. *Behav Res Methods.* **40**, 879–891. 10.3758/BRM.40.3.879 (2008). 18697684 10.3758/brm.40.3.879

[104] Hair, J. F., Ringle, C. M. & Sarstedt, M. PLS-SEM: Indeed a silver bullet. *J. Mark. Theory Pract.* **19**, 139–152. 10.2753/MTP1069-6679190202 (2011).

[105] Ning, H. Research on influencing factors of Museum cultural and creative products purchase intention based on traditional cultural value. In *Master’s thesis, Wuhan University*. 10.27379/d.cnki.gwhdu.2020.001510 (2020).

[106] Jantzen, C., Fitchett, J., Østergaard, P. & Vetner, M. Just for fun? The emotional regime of experiential consumption. *Mark. Theory* **12**(2), 137–154. 10.1177/1470593112441565 (2012).

[107] Yilmaz, T., Sagfossen, S. & Velasco, C. What makes NFTs valuable to consumers? Perceived value drivers associated with NFTs liking, purchasing, and holding. *J. Bus. Res.* **165**, 114056. 10.1016/j.jbusres.2023.114056 (2023).

[108] Zhang, Z. J. Cryptopricing: Whence comes the value for cryptocurrencies and NFTs?. *Int. J. Res. Mark.* **40**, 22–29. 10.1016/j.ijresmar.2022.08.002 (2023).

[109] Dong, Y. & Wang, C. Copyright protection on NFT digital works in the Metaverse. *Secur. Saf.* **2**, 2023013. 10.1051/sands/2023013 (2023).

[110] Çağlayan Aksoy, P. & Özkan Üner, Z. NFTs and copyright: Challenges and opportunities. *J. Intellect. Prop. Law Pract.* **16**, 1115–1126. 10.1093/jiplp/jpab104 (2021).

[111] Yu, W. Theoretical explanation and institutional topology of financial risk regulation for NFT digital collectibles. *Manag. Stud.*10.17265/2328-2185/2022.05.005 (2022).

[112] Yuan, Q. Risk identification and response strategies for the development of digital collections in public libraries from the perspective of the metaverse. *Sichuan Library J*. 62–67. https://www.cnki.com.cn/Article/CJFDTOTAL-TUSH202401009.htm (2024).

[113] Yang, F., Tang, J., Men, J. & Zheng, X. Consumer perceived value and impulse buying behavior on mobile commerce: The moderating effect of social influence. *J. Retail. Consum. Serv.* **63**, 102683. 10.1016/j.jretconser.2021.102683 (2021).

[114] Eagly, A. H. & Chaiken, S. The psychology of attitudes, harcourt brace jovanovich college publishers. *J. Loss Prev. Process Ind.* **8**, 299–305. (1993).

[115] Shelvia, O., Prayitno, A., Rahim, R. & Sundjaja, A. Analysis of Factors Affecting Consumer’s Continuance Intention to Use Mobile Payments with a Value-Based Adoption Model (Vam) Approach. *Psychology and education*,**57**, 2883–2898. (2020).

[116] Tseng, T. H., Wu, T.-Y., Lian, Y.-H. & Zhuang, B.-K. Developing a value-based online learning model to predict learners’ reactions to internet entrepreneurship education: The moderating role of platform type. *Int. J. Manag. Educ*. **21**, 100867. 10.1016/j.ijme.2023.100867 (2023).

[117] Lai, Z. J., Leong, M. K., Khoo, K. L. & Sidhu, S. K. Integrating technology acceptance model and value-based adoption model to determine consumers’ perception of value and intention to adopt AR in online shopping. *Asia Pac. J. Mark. Logist*. **37**, 1–19. 10.1108/APJML-03-2024-0386 (2024).

